# Gene signature of the post-Chernobyl papillary thyroid cancer

**DOI:** 10.1007/s00259-015-3303-3

**Published:** 2016-01-26

**Authors:** Daria Handkiewicz-Junak, Michal Swierniak, Dagmara Rusinek, Małgorzata Oczko-Wojciechowska, Genevieve Dom, Carine Maenhaut, Kristian Unger, Vincent Detours, Tetiana Bogdanova, Geraldine Thomas, Ilya Likhtarov, Roman Jaksik, Malgorzata Kowalska, Ewa Chmielik, Michal Jarzab, Andrzej Swierniak, Barbara Jarzab

**Affiliations:** Department of Nuclear Medicine and Endocrine Oncology, Maria Skłodowska-Curie Memorial Cancer Center and Institute of Oncology, Gliwice Branch, Wybrzeze Armii Krajowej 15, 44-101 Gliwice, Poland; Genomic Medicine, Department of General, Transplant and Liver Surgery, Medical University of Warsaw, Warsaw, Poland; Institute of Interdisciplinary Research, Université libre de Bruxelles (ULB), Bruxelles, Belgium; Human Cancer Studies Group, Division of Surgery and Cancer, Imperial College London Hammersmith Hospital, London, UK; Research Unit Radiation Cytogenetics, Helmholtz-Zentrum, Munich, Germany; Institute of Endocrinology and Metabolism, Kiev, Ukraine; Radiation Protection Institute, Academy of Technological Sciences of Ukraine, Kiev, Ukraine; Systems Engineering Group, Faculty of Automatic Control, Electronics and Informatics, Silesian University of Technology, Gliwice, Poland; Department of Tumour Pathology, Maria Skłodowska-Curie Memorial Cancer Center and Institute of Oncology, Gliwice Branch, Gliwice, Poland; IIIrd Department of Radiation Therapy, Maria Skłodowska-Curie Memorial Cancer Center and Institute of Oncology, Gliwice Branch, Gliwice, Poland; Department of Automatic Control, Silesian University of Technology, Gliwice, Poland

**Keywords:** Papillary thyroid cancer, Children, Adolescents, Radiation, Gene expression, Transcriptome

## Abstract

**Purpose:**

Following the nuclear accidents in Chernobyl and later in Fukushima, the nuclear community has been faced with important issues concerning how to search for and diagnose biological consequences of low-dose internal radiation contamination. Although after the Chernobyl accident an increase in childhood papillary thyroid cancer (PTC) was observed, it is still not clear whether the molecular biology of PTCs associated with low-dose radiation exposure differs from that of sporadic PTC.

**Methods:**

We investigated tissue samples from 65 children/young adults with PTC using DNA microarray (Affymetrix, Human Genome U133 2.0 Plus) with the aim of identifying molecular differences between radiation-induced (exposed to Chernobyl radiation, ECR) and sporadic PTC. All participants were resident in the same region so that confounding factors related to genetics or environment were minimized.

**Results:**

There were small but significant differences in the gene expression profiles between ECR and non-ECR PTC (global test, *p* < 0.01), with 300 differently expressed probe sets (*p* < 0.001) corresponding to 239 genes. Multifactorial analysis of variance showed that besides radiation exposure history, the *BRAF* mutation exhibited independent effects on the PTC expression profile; the histological subset and patient age at diagnosis had negligible effects. Ten genes (*PPME1*, *HDAC11*, *SOCS7*, *CIC*, *THRA*, *ERBB2*, *PPP1R9A*, *HDGF*, *RAD51AP1*, and *CDK1*) from the 19 investigated with quantitative RT-PCR were confirmed as being associated with radiation exposure in an independent, validation set of samples.

**Conclusion:**

Significant, but subtle, differences in gene expression in the post-Chernobyl PTC are associated with previous low-dose radiation exposure.

**Electronic supplementary material:**

The online version of this article (doi:10.1007/s00259-015-3303-3) contains supplementary material, which is available to authorized users.

## Introduction

Following the nuclear accidents in Chernobyl and 25 years later in Fukushima, the nuclear community has been faced with two important issues: first how to manage radiation contamination, and second how to search for and diagnose biological consequences of low-dose internal radiation contamination. The biological consequences of radioiodine contamination after the Chernobyl accident were observed as early as a few years after the accident when an increase in childhood papillary thyroid carcinomas (PTCs) was demonstrated [1, 2]. Since then, approximately 5,000 thyroid cancer cases have occurred in the contaminated regions of Belarus, Ukraine and Russia, with a persisting increased risk of PTC development in irradiated children [3]. Although the increase in PTC incidence in contaminated regions is well demonstrated, it is still not clear whether the molecular phenotype of PTCs associated with low-dose radiation exposure differ from that of sporadic PTC.

In small-scale molecular studies comparing radiation-associated thyroid cancers with sporadic ones in patients of similar age, no differences were observed in the overall frequency of *RET*/*PTC* rearrangements, events crucial for the activation of MAPK cascade [4–12], or in relation to the radiation dose to the thyroid [13]. On the other hand, some studies have shown only distinct types of *RET*/*PTC* rearrangement in patients with radiation-associated and sporadic cancer [10, 11] or a difference between radiation-induced and sporadic PTC using immunohistochemical, genomic and proteomic approaches [14–16]. However, these results could have been biased by many confounding factors (for review see Maenhaut et al. [17]) since, except in one study [15], they were not controlled for the potential impact of genetic and environmental factors, patient age, histological variant or stage of disease.

 Such a well-balanced comparison study was not possible until the establishment of the Chernobyl Tissue Bank (CTB). Since 1998, the CTB (www.chernobyltissuebank.com) has been prospectively collecting samples of thyroid tissue taken from surgical specimens from patients aged under 19 at the time of the Chernobyl accident and resident in the contaminated areas of Ukraine and Russia. The prospective nature of the collection means that it now includes patients with thyroid cancer who were born after the radioactive iodine released from the accident had decayed in the environment. The results of two recent studies using samples provided by the CTB [18, 19] on the gene expression phenotype of PTC developing after low-dose radiation exposure have been reported. However, differences were reported only in normal thyroid tissue [19] or between tumour and normal tissue in relation to radiation dose, but not as global differences [18, 20].

In contrast, this study searched for global differences in molecular profiles in tumour tissue from patients who were either exposed to Chernobyl radiation as children (exposed to Chernobyl radiation, ECR) or were born after 1 January 1987 and therefore not exposed to radiation (not exposed to Chernobyl radiation, non-ECR). Both groups resided in the same areas so that potential confounding factors (e.g. environment) were minimized. Gene expression profiles with respect to intrinsic potential confounding factors including age at PTC diagnosis, mutational status and histological subtype of PTC were also investigated. The study was performed as part of the GENRISK-T project (EU grant FP6 36495) the aim of which is to establish whether individual genetic factors influence the risk of developing cancer of the thyroid after exposure to ionizing radiation.

## Materials and methods

The biological material for gene expression analysis was provided by the CTB as aliquots of total RNA from carefully selected PTC tumour samples paired with RNA extracted from the normal thyroid tissue of the same patient (Supplementary Figure S1), after histopathological review of specimens. After control for RNA and microarray quality, 65 PTC samples were analysed. All biological material was obtained with the informed consent of either the patient or his/her guardian, and following approval of this project by the CTB’s External Review Panel. The CTB samples were supplemented by 24 tumour samples (Supplementary Figure S1) collected from Polish patients with differentiated thyroid cancer (DTC) born between 1 January 1987 and 1994, who were included in the non-ECR group (only for the validation and exon array study). All samples were taken during surgery with the approval by local Ethics Committee and informed consent was obtained from all patients.

### 3′ Oligonucleotide microarray study

The study included 33 PTC samples from the ECR group and 32 samples from the non-ECR group, all obtained from the CTB. In the ECR group, the patients received a mean thyroid radiation dose of 288 mGy (range 45.4 to 4,595). In seven patients (21 %) the radiation dose was higher than 1 Gy and only in five (15 %) lower than 100 mGy. At the time of PTC diagnosis, the ECR patients were slightly but significantly older than the non-ECR patients. There were no significant differences with respect to the histological subtype. The distribution of other factors, especially disease stage was comparable between the two groups (Table 1).

**Table 1:** Comparison of ECR and non-ECR patents included in the microarray study and in an independent qPCR validation study

		Microarray study set		qPCR validation set			*p* value (microarray vs. validation set)		
		ECR	non-ECR	*p* value	ECR	non-ECR	*p* value	ECR	non-ECR
Number		33	32	ND	19	17	ND	ND	ND
Female/male		23/10	26/6	NS	14/5	14/3	NS	NS	NS
Age at exposure (years), median (range)		2.3 (0.1 – 8.3)	ND	ND	2 (0.5 – 11.2)	ND	ND	NS	ND
Age at diagnosis (years), median (range)		17.7 (14.7 – 24.5)	16.3(7.7 – 21)	**0.0002**	19.5 (1.3 – 23.9)	17.4 (11.6 – 21.5)	0.06	0.07	**0.04**
Place of residence (province)									
Kiev		10 (30.3 %)	12 (37.5 %)	NS	7 (36 %)	–^b^	ND	NS	ND
Zhytomir		8 (24.2 %)	5 (15.6 %)	NS	5 (26 %)		ND	NS	ND
Chernigow		8 (24.2 %)	5 (15.6 %)	NS	6 (31.5)		ND	NS	ND
Sumy, Rovno, Chercassy, Pipriad		7 (21.2 %)	10 (32.3 %)	NS	1 (5 %)		ND	NS	ND
Histopathology^a^									
Pure classic PTC		4 (12 %)	7 (22 %)	NS	4 (21.1 %)	12 (70.6 %)	ND	NS	ND
PTC with follicular areas		17 (52 %)	11 (33 %)	NS	6 (31.6 %)	5 (29.4 %)	ND	NS	ND
PTC with solid areas		12 (36 %)	14 (43 %)	NS	8 (42 %)	0	ND	ND	ND
Unknown		0	0	ND	1 (5.2 %)	0	ND	ND	ND
Mutational status of PTC									
RET/PTC rearrangements	Positive	10 (30.3 %)	8 (25 %)	NS	Not available		ND		
	Negative	19 (57.6 %)	22 (66.7 %)	NS					
	Unknown	4 (12.1 %)	2 (6.3 %)	NS					
BRAF V600E mutation	Positive	8 (24.2)	6 (18.8 %)	NS					
	Negative	22 (66.7 %)	21 (65.6 %)	NS					
	Unknown	3 (9.1 %)	5 (15.6 %)	NS					
Primary tumour (T stage)									
1		12 (36 %)	11 (34 %)	NS	3 (15.8 %)	8 (47.1 %)	NS	NS	
2		4 (12 %)	5 (16 %)	NS	4 (21.1 %)	4 (23.5 %)	NS	NS	
3		17 (52 %)	16 (50 %)	NS	11 (57.9 %)	5 (29.4 %)	NS	NS	
Unknown		0	0	–	1 (5.2 %)	0	–		
Lymph nodes (N stage)									
0		14 (42 %)	14 (34 %)	NS	8 (42.1 %)	6 (35.3 %)	NS	NS	
1		19 (58 %)	18 (56 %)	NS	10 (57.9 %)	11 (64.7 %)	NS	NS	
1a		12 (36 %)	8 (25 %)	NS	7 (36.9 %)	4 (23.5 %)	NS	NS	
1b		7 (22 %)	10 (31 %)	NS	3 (15.8 %)	7 (41.2 %)	NS	NS	
Unknown		0	0		1	0			
Distant metastases (M stage)									
0		29 (87 %)	30 (94 %)	NS	17 (89.3 %)	16 (94.1 %)	NS	NS	
1		4 (13 %)	2 (6 %)	NS	2 (10.7 %)	1 (5.9 %)	NS	NS	

*ND* not determined, *NS* not significant

Significant *p* values are shown in bold

^a^In the ECR groups and non-ECR group in the microarray study, histopathology was evaluated according the CTB criteria as: pure classic PTC, PTC with follicular areas (dominant pattern of follicular structures), and PTC with solid areas (dominant pattern of solid areas). In the non-ECR group from Poland (qPCR validation study), histopathology was evaluated according to the WHO 2004 criteria

^b^Group from Poland

Details on RNA isolation and microarray analysis are provided in the Supplementary material.

### Validation qPCR study

In a validation study 19 independent samples from ECR patients from the CTB were compared with 17 samples from non-ECR Polish patients. Since CTB did not posses additional non-ECR tumour samples we decided to include samples from Polish patients because of the common ethnicity of Ukrainian and Polish children and stable iodine prophylaxis in Poland after the Chernobyl accident that resulted in a stable incidence of childhood DTC. The Polish patients were selected to ensure their common ethnicity profile with the CTB patients. The characteristics of the validation group are presented in Table 1.

Details of the qPCR analysis are provided in the Supplementary material.

### Validation exon array study

An additional comparison of the exon expression profiles was performed for 27 PTC patients under the age of 26 years, 13 ECR and 14 non-ECR (Supplementary Table S1). Twenty RNA samples were derived from PTC patients previously included in the initial 3′ microarray study and from seven new PTC patients of whom six were Polish born during the period 1981 – 1992. Expression analysis of all human exons was carried out using an Affymetrix Human Exon 1.0 ST array.

### Data analysis

The data discussed in this article have been deposited in NCBI’s Gene Expression Omnibus [21] and are accessible through GEO series accession number GSE35570 (http://www.ncbi.nlm.nih.gov/geo/query/acc.cgi?acc=GSE35570).

Microarray data were normalized using the GCRMA algorithm. First multidimensional scaling was performed. Then we applied a method of our own for gene filtering based on a comprehensive analysis of the technical accuracy of the measurement of thyroid cancer and normal thyroid tissue gene expression by oligonucleotide microarrays done on the same samples in two independent laboratories at the Université libre de Bruxelles, Belgium, and the Institute of Oncology in Gliwice, Poland. Briefly, a subset of 19 Affymetrix CEL files was analysed in both laboratories, Bruxelles and Gliwice, and the results from the two laboratories for the same tumour and normal tissue samples were compared. The overall correlations between the results for pairs of samples from the two laboratories were excellent (0.982 – 0.994). However, while analysing the transcript-by-transcript correlations, we observed that only a subpopulation of probe sets showed excellent reproducibility. There was a trend for an increasing correlation with increasing expression level and variance. After extensive analysis of these relationships, the dataset was subdivided into sets of probe sets according to their expression and variance to discriminate between sets with good, acceptable and poor correlation. Genes showing poor reproducibility (log2 mean expression less than 5 and variance less than the upper quartile of the variances of all probe sets) were filtered out before the final analysis.

Genes differentially expressed between the ECR and non-ECR groups were selected by a randomized block design with two microarray batches. We used a noncorrected threshold of *p* < 0.001. A global test was applied to assess the overall significance of the result, the Benjamini-Hochberg false discovery rate (FDR) was calculated for every transcript.

Functional enrichment analysis, including the Kyoto Encyclopaedia of Genes and Genomes database (KEGG; www.genome.jp/kegg/) and Panther pathway software (www.pantherdb.org/), were performed to identify metabolic pathways and groups of genes with similar metabolic function based on their annotation. A Bonferroni-corrected *P* value of 0.05 was considered statistically significant. The effects of putative confounding factors such as age at PTC diagnosis, presence of solid histoarchitecture, and presence of *BRAF* or *RET*/*PTC* alteration, were analysed by separated three-way analysis of variance (ANOVA) using batches as the fourth, blocking, factor. Part of the analysis was performed using BRB-ArrayTools developed by Dr. Richard Simon and the BRB-ArrayTools Development Team (http://linus.nci.nih.gov/BRB-ArrayTools.html).

## Results

### Differences between ECR and non-ECR papillary thyroid cancer

**Fig. 1:**
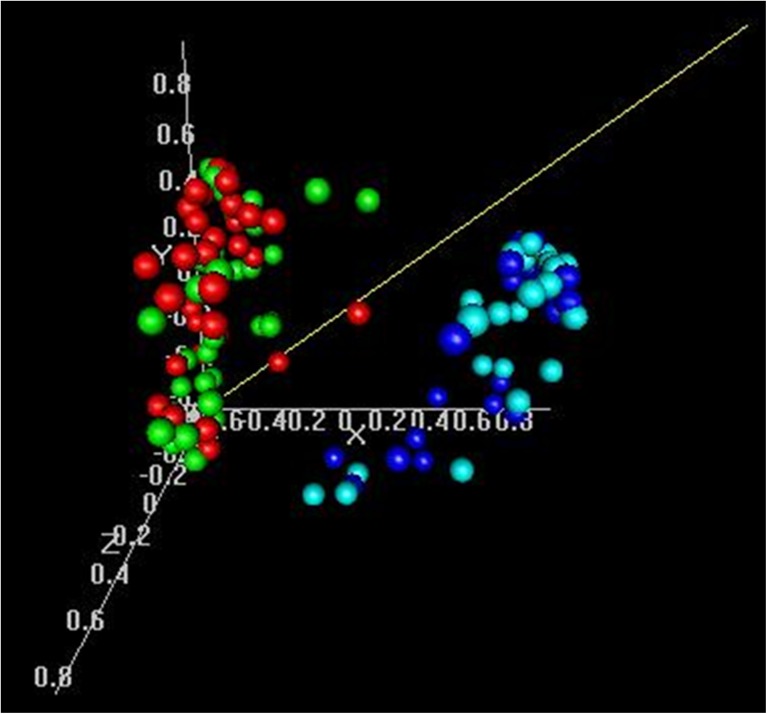
Multidimensional scaling of samples. Samples coloured *red* are ECR tumours, *green* are non-ECR tumours, *blue* are ECR normal thyroids, and *cyan* are non-ECR normal thyroids

The unsupervised multidimensional scaling analysis showed no global difference in expression between ECR and non-ECR tumours (Fig. 1). However, after filtering out the low-reproducibility probe sets, with stratification for two batches, 300 probe sets were differentially expressed between ECR and non-ECR tumours (noncorrected *p* < 0.001 with FDR for these genes in the range 0.5 – 8.5 %), and this difference was significant (*p* < 0.01) in the global test of difference as implemented in BRB-ArrayTools, i.e. this number of genes was not likely to be obtained by chance. These 300 transcripts corresponded to 239 known genes (Table 2 and Supplementary Table S2 and Fig. S2). Pathway enrichment analysis in the KEGG database showed that genes differentially expressed between ECR and non-ECR tumours were involved in two endocrine-related cancer pathways (prostate and endometrium), non-small-cell lung cancer and tight junction. In the Panther pathway analysis among others the PI3 kinase pathway was involved (Supplementary Table S3).

**Table 2:** Top 30 genes differentiating ECR and non-ECR papillary thyroid cancers

Gene		ECR/non-ECR expression microarray		ECR/non-ECR 3-ANOVA		ECR/non-ECR exon microarray		ECR/non-ECR qPCR	
Symbol	Description	FDR	Fold change	P value	FDR	FDR	Fold change	FDR	Fold change
*USP27X*	Ubiquitin-specific peptidase 27, X-linked	0.00516	1.34	0.0000093	0.05	NS		ND	
*ANKS6*	Ankyrin repeat and sterile alpha motif domain containing 6	0.0103	1.34	0.0000152	0.05	0.058	1.35	ND	
*GPX7*	Glutathione peroxidase 7	0.0103	0.61	0.0000007	0.019	9		NS	
*MNT*	MAX binding protein	0.0187	1.28	0.0000031	0.04	0.058	1.22	NS	
*PPP1R9A*	Protein phosphatase 1, regulatory (inhibitor) subunit 9A	0.0341	1.5	0.0000137	0.05	NS		0.021	1.225
*MKNK2*	MAP kinase interacting serine/threonine kinase 2	0.0341	1.29	0.000114	0.094	0.133	1.22	NS	
*DDR1*	Discoidin domain receptor tyrosine kinase 1	0.0341	1.34	0.000353	NS	0.070	1.33	ND	
*HNRNPUL2*	Heterogeneous nuclear ribonucleoprotein U-like 2	0.0341	1.25	0.0000225	0.053	0.070	1.08	ND	
*GNL1*	Guanine nucleotide binding protein-like 1	0.0341	1.23	0.000235	NS	0.101	1.12	ND	
*PTCD3*	Pentatricopeptide repeat domain 3	0.0341	0.8	0.0000205	0.053	NS		ND	
*ZBTB43*	Zinc finger and BTB domain containing 43	0.0341	1.4	0.000068	0.09	NS		ND	
*CIC*	Capicua homologue (*Drosophila*)	0.0341	1.26	0.0000521	0.09	NS		0.008	1.221
*GMEB2*	Glucocorticoid modulatory element binding protein 2	0.0341	1.26	0.000213	NS	0.070	1.18	ND	
*ZBTB7C*	Zinc finger and BTB domain containing 7C	0.0341	1.44	0.00015	NS	0.148	1.22	ND	
*KIAA0182*	KIAA0182	0.0341	1.32	0.000311	NS	0.123	1.14	ND	
*GNA11*	Guanine nucleotide binding protein (G protein), alpha 11 (Gq class)	0.0341	1.25	0.000112	0.094	0.130	1.22	NS	
*HDAC11*	Histone deacetylase 11	0.0341	1.47	0.000071	0.09	0.114	1.20	0.004	1.247
*SPATA2L*	Spermatogenesis associated 2-like	0.0341	1.26	0.000695	NS	NS		ND	
*SLC25A23*	Solute carrier family 25 (mitochondrial carrier, phosphate carrier), member 23	0.0341	1.41	0.000126	NS	0.090	1.31	ND	
*TIA1*	TIA1 cytotoxic granule-associated RNA binding protein	0.0341	0.73	0.0000142	0.05	NS		ND	
*PALM3*	Paralemmin-3	0.0341	3.42	0.000182	NS	0.136	1.17	NS	
*LYPLA2*	Lysophospholipase II	0.0341	1.23	0.000138	NS	NS		ND	
*MOB2*	Mps one binder kinase activator-like 2	0.0341	1.22	0.000189	NS	NS		ND	
*HDGF*	hepatoma-derived growth factor (high-mobility group protein 1-like)	0.0349	1.19	0.0000155	0.05	0.070	1.13	0.021	1.275
*GOPC*	Golgi-associated PDZ and coiled-coil motif containing	0.0359	1.36	0.0000616	0.09	NS		ND	
*JUB*	Jub, ajuba homolog (*Xenopus laevis*)	0.0359	1.39	0.000181	NS	0.093	1.25	NS	
*CTBP2*	C-terminal binding protein 2	0.0359	1.29	0.000171	NS	NS		ND	
*EHMT2*	Euchromatic histone-lysine *N*-methyltransferase 2	0.0362	1.27	0.00011	0.094	0.101	1.17	ND	
*RAD51AP1*	RAD51 associated protein 1	0.0362	0.58	0.000218	NS	0.100	0.86	0.021	0.553
*SPRYD3*	SPRY domain containing 3	0.0362	1.3	0.000672	NS	0.075	1.32	ND	

*FDR* false discovery rate, *ND* not determined, *NS* not significant

### Analysis of potential confounding factors

In-depth analysis of the potential intrinsic confounding factors was carried out to exclude their influence on the radiation-related differences in gene expression profile. Initially, age at PTC diagnosis, presence of solid pathomorphology and presence of the *BRAF* or *RET*/*PTC* alteration known to trigger PTC were considered for their relationship with the differences in gene expression between ECR and non-ECR tumours by separate three-way analyses of variance (Supplementary Table S4). No association between gene expression profile and patient age (younger than 16 years of age vs. older) was seen for a FDR of <10 %. *BRAF* mutation was significantly associated with the PTC gene expression profile (794 probe sets), while there were only 13 probe sets associated with *RET*/*PTC* rearrangement with the same criteria. The difference in gene expression related to radiation exposure was also independently significant in the presence of solid pathomorphology. In the final analysis of putative confounding factors, we included *BRAF* mutation and solid PTC variant with radiation exposure. Our analysis revealed that radiation exposure was associated with differences in gene expression regardless of the *BRAF* mutation effect (significantly associated with a number of transcripts) and of the influence of solid PTC variant, that was negligible in multivariate analysis for a FDR <10 % (Table 3).

**Table 3:** The gene signature of exposure to Chernobyl radiation: analysis of putative confounding factors. First, four different three-way analyses were performed (with series-related subgroups) for interaction with age at diagnosis, presence of the *BRAF* or *RET*/*PTC* alteration, and solid histoarchitecture. For each of the analysed factors, the number of genes significant at *p* < 0.001 is shown in Supplementary Table S4. A final analysis performed for the three factors with the strongest effect and two series of examinations, which included exposure to Chernobyl radiation, *BRAF* mutation, and pathology (with subdivision into two groups, one including the classic and follicular variants, and the other both specified subgroups with solid appearance) is shown

Effect	No of probe sets at *p* < 0.001	No of probe sets at FDR <10 %
Exposure to Chernobyl radiation	196	33
*BRAF* mutation	183	114
Pathology (classical and follicular/solid component)	32	0

### Validation of the results by qPCR

**Fig. 2:**
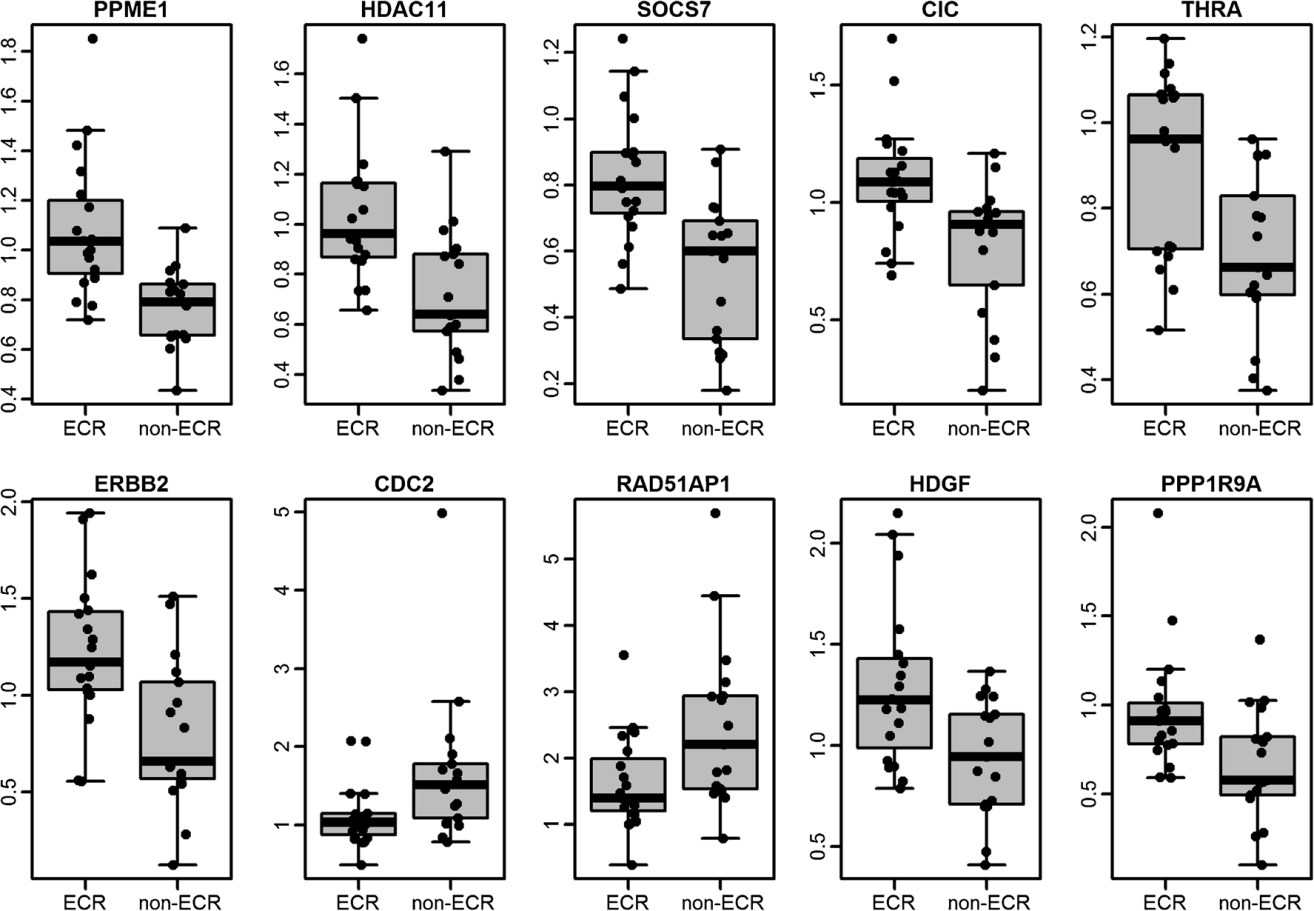
Genes validated in the qPCR study

To validate the low-dose irradiation-induced changes in gene expression, we selected 19 genes from the ECR/non-ECR gene signature for qPCR in an independent set of 36 PTC. In the ECR group there were 19 PTC samples derived from CTB and independent of the microarray set, and in the non-ECR group 17 samples collected from adolescent Polish patients undergoing surgery because of PTC (Supplementary Figure S1). Gene selection was performed based on preliminary microarray analysis (data not shown). The criterion for selection was a significant difference in expression between ECR and non-ECR tumours, and the biological function of the gene (we decided to select genes involved in response to DNA damage). The curated list of 19 genes was selected (Supplementary Table S5) and expression of all of them was estimated by qPCR independent from the set of PTC samples, separate from those investigated by microarray. Of the 19 genes, 10 (52 %) were validated: *PPME1* (fold changes of 1.19 and 1.25 in the ECR group in the microarray experiment and in qPCR validation, respectively), *HDAC11* (fold changes of 1.47 and 1.25), *SOCS7* (fold changes of 1.38 and 1.22), *CIC* (fold changes of 1.26 and 1.22), *THRA* (fold changes of 1.32 and 1.16), *ERBB2* (fold changes of 1.32 and 1.34), *PPP1R9A* (fold changes of 1.5 and 1.23), *HDGF* (fold changes of 1.19 and 1.28), *RAD51AP1* (fold changes of 0.58 and 0.55) and *CDK1* (fold changes of 0.57 and 0.67) (Fig. 2). Genes that were not confirmed in the qPCR analysis included *MKNK2*, *RAS*, *JUB*, *USP15*, *FAM105A*, *MNT*, *GPX7*, *PALM3* and *GNA11*.

### Validation by exon array

Finally, 27 PTC (13 ECR, and 14 non-ECR) were considered for the exon expression validation analysis (in the non-ECR group there were eight CTB samples and eight Polish samples). From the 239 genes specified by the initial gene expression microarray study, 52 (22 %) were confirmed at the level of FDR <10 % (Table 2 and Supplementary Table S2).

## Discussion

Although a rise in the incidence of thyroid cancer after the Chernobyl accident is evident [3, 22], the question of the potential molecular peculiarities of these induced tumours has not yet been resolved. Answering this question is not only of scientific interest, but also may expand our knowledge on how to manage internal radiation contamination.

In our study of post-Chernobyl PTC, we observed small but significant changes in the expression of 239 genes (*p* < 0.01) between tumours arising after exposure to low-dose radiation after the Chernobyl accident and sporadic PTCs. Our study is among the first to find differences in gene expression profiles between radiation-induced and sporadic PTC in patients matched for their ethnicity, place of residence, sex, histopathology, disease stage and age at diagnosis. Five previous transcriptomic studies comparing radiation-induced and sporadic thyroid cancer [16, 23–26] were limited by the small number of studied patients, were not matched between sporadic and radiation-induced PTC due to differences in geographical distribution of the patients [23, 25, 26] and in PTC stage [25], and compared expression alterations in radiation-induced cancer with data repositories of sporadic PTC in adults [16]. The recently published study by Abend et al. [20] in which a well-characterized cohort of patients with radiation-induced PTC were analysed, showed radiation dose-dependent gene expression changes, but did not globally compare exposed and non-exposed patients. Our results support their general conclusion on the long-term differential gene expression in PTC arising after ionizing radiation exposure. This observation is also supported by recent results [15] demonstrating that PTC driver alterations are more prevalent in PTC in children who have been exposed to radiation.

Although to our knowledge our matched group of radiation-exposed patients and patients with sporadic PTC is optimal because of the availability of current biological samples, we are aware of a potential drawback in the ability to identify sporadic PTC developing in radiation-exposed patients. According to epidemiological estimation, about 29 % of patients in our ECR group may have developed PTC in the absence of radiation exposure [27]. The figure may possibly be even higher if the increased identification of PTC due to screening of the population is taken into account. We therefore cannot rule out the inclusion of some sporadic cancers in our ECR group. However, we were able to identify significant, although subtle, differences in gene expression profiles between ECR and non-ECR cancers. We can speculate that the inclusion of sporadic PTC may be one of the reasons for very subtle difference in gene expression with a fold change in the range 0.48 – 3.42. Also at the molecular level, in the ECR group we failed to separate tumours clustering closer to those in the non-ECR group either in the unsupervised multidimensional scaling principle component analysis (PCA) (Fig. 1) or in the more detailed supervised analysis. This leads us to speculate that the different gene expression between ECR and non-ECR tumours is rather related to radiation response than to carcinogenesis.

Our negative findings using PCA are in line with the results of Dom et al. [19], who in cooperation and in parallel with our group studied gene expression in normal thyroid tissue of radiation-exposed and non-exposed patients. They also were not able to show any differences using PCA, and only significance analysis of microarrays (SAM) with adjustment for age was able to identify 403 differentially expressed genes in normal thyroid tissue. Similarly in our study the difference between ECR and non-ECR tumours could only be detected after careful quality assurance, including gene filtering according to their expression level and variance. Thus, with such a stringent criterion, it is not surprising that there were only a few overlapping genes when we compared our 239 differentiating genes with the results of others. None of the top 15 candidate genes found to differ between radiation-induced and sporadic PTC by Port et al. [25] overlapped with ours or two other sets. No overlap was found either for the ten genes validated by us by qPCR. Only one gene (*NEDD4L*) identified by Detours et al. [23], four genes (*ALDH6A1*, *TPD52L1*, *GPX1*, *ECE1*) identified by Stein et al. [16] and two genes (*MYO1C*, *IGF1R*) identified by Ugolin et al. [26] were observed in our microarray gene signature.

 Given that our multifactorial analysis of variance excluded the contribution of age differences and tumour pathology to the difference in gene expression profiles between the ECR group and non-ECR group, one can hypothesize that the genes identified here reflect a true difference between non-ECR and ECR PTC. However, our results also support an independent effect of a *BRAF* mutation on PTC gene expression profile. Interestingly, the effects of the presence of *RET*/*PTC* rearrangements were smaller [28]. This is consistent with the findings of previous studies showing differences between the effects of *BRAF* and *RET*/*PTC* alterations on gene expression in thyroid cancer [29, 30]. The frequency of *RET*/*PTC* rearrangements was not as high, and of *BRAF* mutation not as low as previously reported in post-Chernobyl PTC [7, 8, 12, 31]. This is also consistent with the fact that the median age of the patients at diagnosis was 17.7 years, which is distinctly higher than in previous post-Chernobyl cohorts [3], but similar to the age of the patients recently reported by Sassolas et al. [32]. The relationship between age at diagnosis and frequency of *BRAF* and *RET*/*PTC* alterations has also been previously identified in Ukrainian patients [33]. The requirement to age-match the patients with patients with sporadic PTC, which is more common in older children, in this study meant that patients in the ECR group were also slightly older than in the previous studies that did not use age-matched controls. In addition, 52 of our genes were validated by exon array analysis done in the partially independent and smaller set of tumours.

Environmental factors, such as differences in iodine deficiency, also need to be taken into consideration [34]. However in our study the place of residence of patients in the ECR and non-ECR groups were evenly distributed within different regions (oblast) and we consider that in a retrospective series cases this is the best available method to control for differences in iodine dietary status. Unlike other authors [18, 20], we did not show formal analysis of gene expression in relation to individual radiation doses provided by the CTB [35]. Although the Spearman’s dose–response correlation indicated a few significant genes (data not shown), due to uncertainty in radiation dose and possible inclusion of patients with sporadic PTC in the non-ECR group, we consider these data too biased. Furthermore, recently reported studies indicate more diverse gene expression profile with decreasing absorbed doses. This was observed in mouse thyroid cells after injection of different amounts of ^211^At or ^131^I radionuclides [36, 37]. It was hypothesized that at high absorbed doses, the DNA lesions might have been too complex to be properly repaired, resulting in reduced cellular response compared to that at lower absorbed doses.

An important feature of the investigated PTC patients was their young age, which contributed to the different PTC gene expression profile compared with adult patients (data not shown). However, our radiation gene signature contained both genes, which did and did not contribute to the tumour/normal difference in the studied patients (data not shown) [38, 39]. Thus, our study defined the difference in gene expression related to radiation exposure, and the functional consequences of this need to be defined. To understand the underlying biological mechanisms, the genes confirmed by qPCR need to be examined in independent PTC cases in relation to G2/M cell cycle arrest. The simultaneous lower expression of *CDK1* and *RAD51AP* may represent impaired repair of the radiation-induced DNA damage in ECR patients. The expression of *CDK1* in fibroblasts is reduced in response to radiation [40], and its suppression is essential for DNA damage-induced G2 arrest [41]. *CDK1* is required for efficient 5′ to 3′ resection of double-strand break ends, and for the recruitment of the single-stranded DNA-binding complex, RPA and the RAD51 recombination protein [42]. Decreased *RAD51AP*, encoding an enhancer of *RAD51*, observed in tumours from ECR patients is consistent with this suggestion, as genetic ablation of *RAD51AP1* leads to enhanced sensitivity to chromosome aberrations upon DNA damage [43]. *RAD51AP1*-depleted cells have deficits in recombination-based repair of a DNA double-strand break, and exhibit chromatin breaks both spontaneously and upon DNA-damaging treatment [44].

The simultaneous increase in expression of *HDAC11* in ECR-related PTC creates a link to transcriptional repression and epigenetic landscaping [45], and can be interpreted as concordant with both *CDK1* and *RAD51AP1* decreases as the latter is regulated by E2F family of transcription factors, while histone deacetylases interact with RB-E2F to inhibit gene transcription and are activated by radiation [46]. This effect may be stronger at the basic higher gene expression level. The reduced expression of *PPME1* may also be related to the repair of gamma irradiation-induced DNA damage, which is regulated not only by PP1, but also by PP2A phosphatase inhibition [47]. Its protein product, protein phosphatase methylesterase 1, is regarded as a key molecule that sustains the activation of ERK activity in cancer cells via inhibition of PP2A [47, 48]. The higher expression of this gene group in thyroid cancers of the ECR group may lead to the higher activation of MAP cascade downstream of growth factors, but upstream of RAF and facilitate neoplastic transformation towards PTC [10]. Indeed, 4 of 14 genes known to modulate *PP2A* were significantly changed in ECR-related tumours (Supplementary Table S2). These effects may have been further enhanced be upregulation of *ERBB2* and THRA (thyroid hormone receptor A) in the ECR group. Recently *THRA*-rs939348 was confirmed as a risk factor for DTC [49], and one may speculate that its increased expression in ECR tumours is a persistent response to radiation DNA damage which may cooperate with other genes in DTC development.

Obviously, a number of other potential speculative explanations for the observed gene expression differences could be presented. It cannot be excluded that cancer induced by a single dose of radiation shows a difference in cellular homogeneity (increased number of multiplied transformed cells and their desynchronization), kinetics of progression, or even in the tumour size at diagnosis.

An important study of the molecular biology of thyroid cancer discussing the results of The Cancer Genome Atlas (TCGA) has recently been published [28]. The results of the study indicate the relatively low number of novel genomic events in PTC compared to the previous knowledge and indicate the presence of subtypes, mainly related to the type of initiating somatic abnormality. It is an obvious next step to apply genomic sequencing to analyse in-depth the association of these subtypes and heterogeneity related to different initiating mutations with the profile of radiation-induced PTC. It is important to note that the expression of all genes characteristic of ECR PTCs according to our signature in the PTCs investigated by TCGA was high.

The important question arises as to whether subtle differences between the profiles of radiation-induced and sporadic PTCs have any clinical significance. Probably they do not reflect profound differences in the underlying disease, but rather different disease kinetics, cellular composition or – most interestingly – additional molecular mechanisms operating in the radiation-induced cancer. The proposed classifier is not sufficient in itself to distinguish the cancers induced by low-dose radiation from sporadic cancers, and our results indicate that the effect of radiation is similar in scale to many other factors influencing the variability of gene expression in PTC. We did not find any gene expression differences profound enough to influence the clinical course of the disease, and this is in line with the clinical observations indicating similar prognosis in post-Chernobyl childhood PTC [3, 50]. However, we interpret the differences observed by us as an excellent starting point to assess the importance of genes constituting the radiation signature in the pathogenesis of PTC.

In conclusion, we report significant, but subtle, differences in gene expression in the post-Chernobyl PTC that are associated with low-dose radiation exposure. Since the population exposed to low-dose thyroid radiation (either medical or accidental) is increasing, the study may serve as a basis for further studies on the susceptibility of the thyroid to low-dose radiation.

## Electronic supplementary material

Below is the link to the electronic supplementary material.

ESM 1 (DOC 22 kb)

Fig. S1 (PDF 727 kb)

Fig. S2 (PDF 86 kb)

Table S1 (PDF 72 kb)

Table S2 (XLS 70 kb)

Table S3 (XLS 21 kb)

Table S4 (PDF 14 kb)

Table S5 (PDF 344 kb)
