# Supplementary material for: Gene signature of the post-Chernobyl papillary thyroid cancer
Source: Eur J Nucl Med Mol Imaging. 2016 Jan 26;43:1267–77. doi: 10.1007/s00259-015-3303-3 (PMC4869750; doi:10.1007/s00259-015-3303-3)
Supplement: Supplementary file 1 — (DOC 22 kb) [file 259_2015_3303_MOESM1_ESM.doc]

**Supplementary Material and Methods.**

***RNA isolation.*** Total RNA from the CTB was obtained by homogenization of frozen tissue using Tissuelyser II (Qiagen GmbH, Hilden, Germany) followed by extraction and purification using RNeasy Mini Kits (Qiagen).RNA quality was estimated by Agilent 2100 using RNA 6000 Nano Assay (Agilent Technologies Santa Clara CA, United States). RNA integrity, assessed by RNA Integrity Number (RIN) index (Agilent), was within the range of 6.4–9.3 for Ukrainian samples, and within the range of 6.9–8.5 for Polish tumors. RNA quantity was measured by NanoDrop ND-1000 minispectrophotometer.

***3’ oligonucleotide gene expression analysis.*** Only samples with RIN > 7 were included to microarray analysis, resulting in 136 samples with sufficient RNA quality/quantity. Microarray analysis was performed according to recommendations of Affymetrix Gene Expression Analysis Technical Manual (Santa Clara, CA, United States). Briefly, 4 µg (first batch of 53 samples) or 2 µg (second batch of 76 samples) was used as a template for cDNA synthesis (One-Cycle cDNA Synthesis Kit, Affymetrix), and further in vitro transcription step was performed using IVT Labeling Kit (Affymetrix). Labeled cRNA was purified by GeneChip Sample Cleanup Module, and the quality of biotinylated cRNA was evaluated by capillary electrophoresis (Bioanalyzer 2100, Agilent) and then fragmented and hybridized to Human Genome U133 2. Plus 2.0 array (Affymetrix). After washing and staining with streptavidin-phycoerythrin conjugate arrays were scanned in GeneChip 3000G scanner (Affymetrix). Arraying procedures were carried out in two batches, batch was further used as a stratification variable.

***qPCR study.*** Total RNA from Polish samples was extracted from homogenized frozen tissue using RNeasy Micro Kits (Qiagen), which also included a DNA-se I digestion step. Quantitative real-time reverse transcription-PCR (qPCR) was carried out with fluorescent probes (Universal Probe Library, Roche Basel, Switzerland). Amplicons were designed using a web-based application (www.roche-applied-science.com/sis/rtpcr/upl). Additionally, amplicons for tested genes were analyzed by BLAST (to ensure that the correct sequence is being amplified and to check for the presence of SNPs). QPCR was carried out in a 96-well optical reaction plate using an ABI Prism 7900HT machine (Applied Biosystems, Life Technologies, Carlsbad CA, USA). Five microliters of template cDNA (equivalent to 500 ng of total RNA) were added to 15 µl of PCR reaction mix containing 10 µl TaqMan Universal PCR Master Mix (Applied Biosystems), 1 µl forward and reverse primers (200 nM), 1 µl probe (100 nM), and water. Thermal cycling conditions were as follows: 50 ºC for 2 min (incubation and activation AmpErase UNG), 95 ºC for 10 min (activation AmpliTag Polymerase DNA), 95 ºC for 15 sec (denaturation), and 60 ºC for 1 min (annealing and extension). Every sample was examined in duplicates. RNA pooled from 5 samples of PTC was used in real-time PCR as a calibrator. Quantitative real-time PCR data were analyzed, normalizing for reaction efficiency and reference index, based on the expression of 4 genes: *ATP6V1E1*, *EIF3S10*, *HADHA*, and *UBE2D2* obtained by analysis using geNorm software.
