# Supplementary material for: Gene signature of the post-Chernobyl papillary thyroid cancer
Source: Eur J Nucl Med Mol Imaging. 2016 Jan 26;43:1267–77. doi: 10.1007/s00259-015-3303-3 (PMC4869750; doi:10.1007/s00259-015-3303-3)
Supplement: Supplementary file 2 — (PDF 727 kb) [file 259_2015_3303_MOESM2_ESM.pdf]

Figure S1. Origin of thyroid samples used in our study
A: gene 3'expression microarray study; B: validation studies (exon microarray and qPCR)

A.

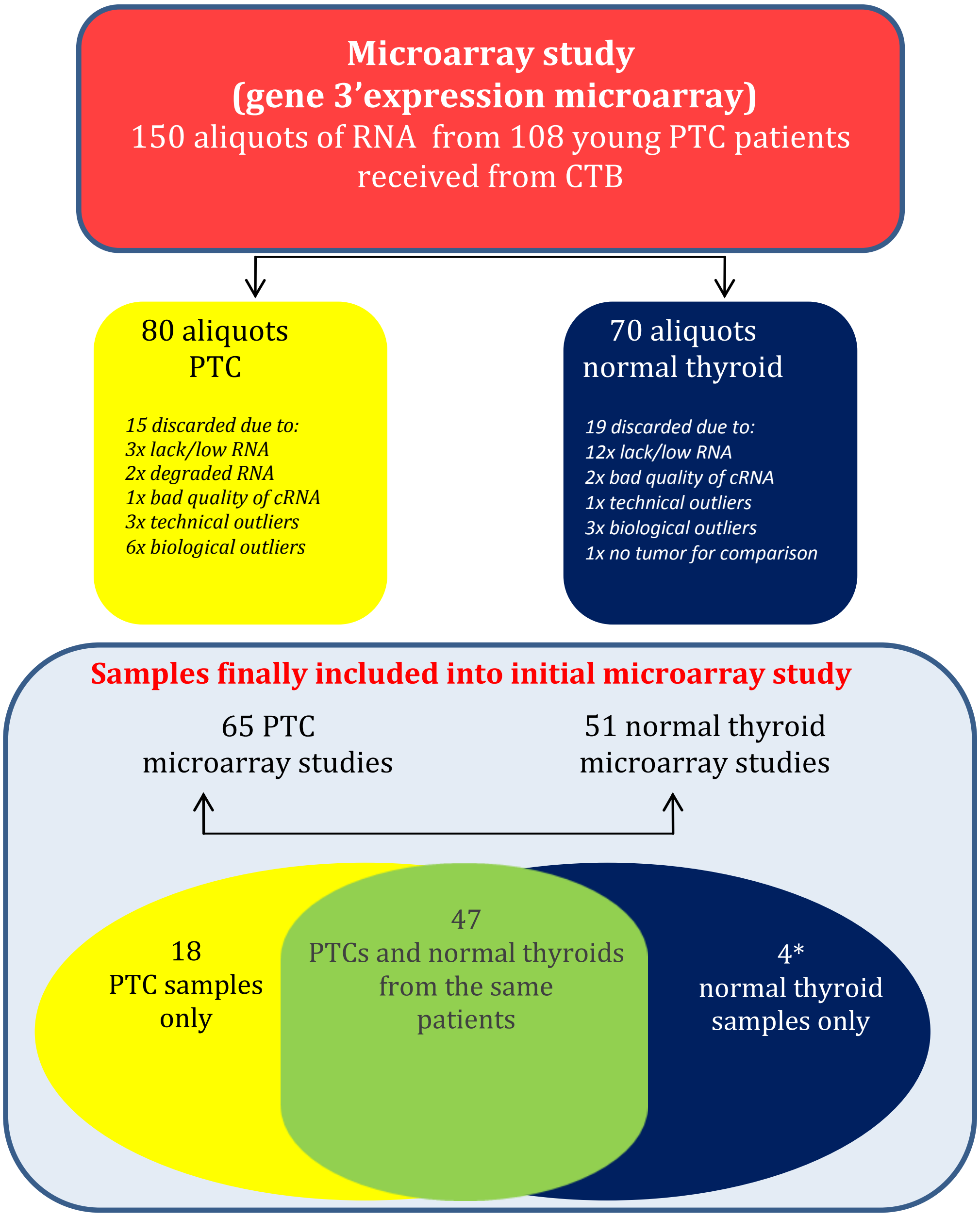

* Their tumor counterparts were discarded (1x lack of RNA; 3x biological outliers)

B.

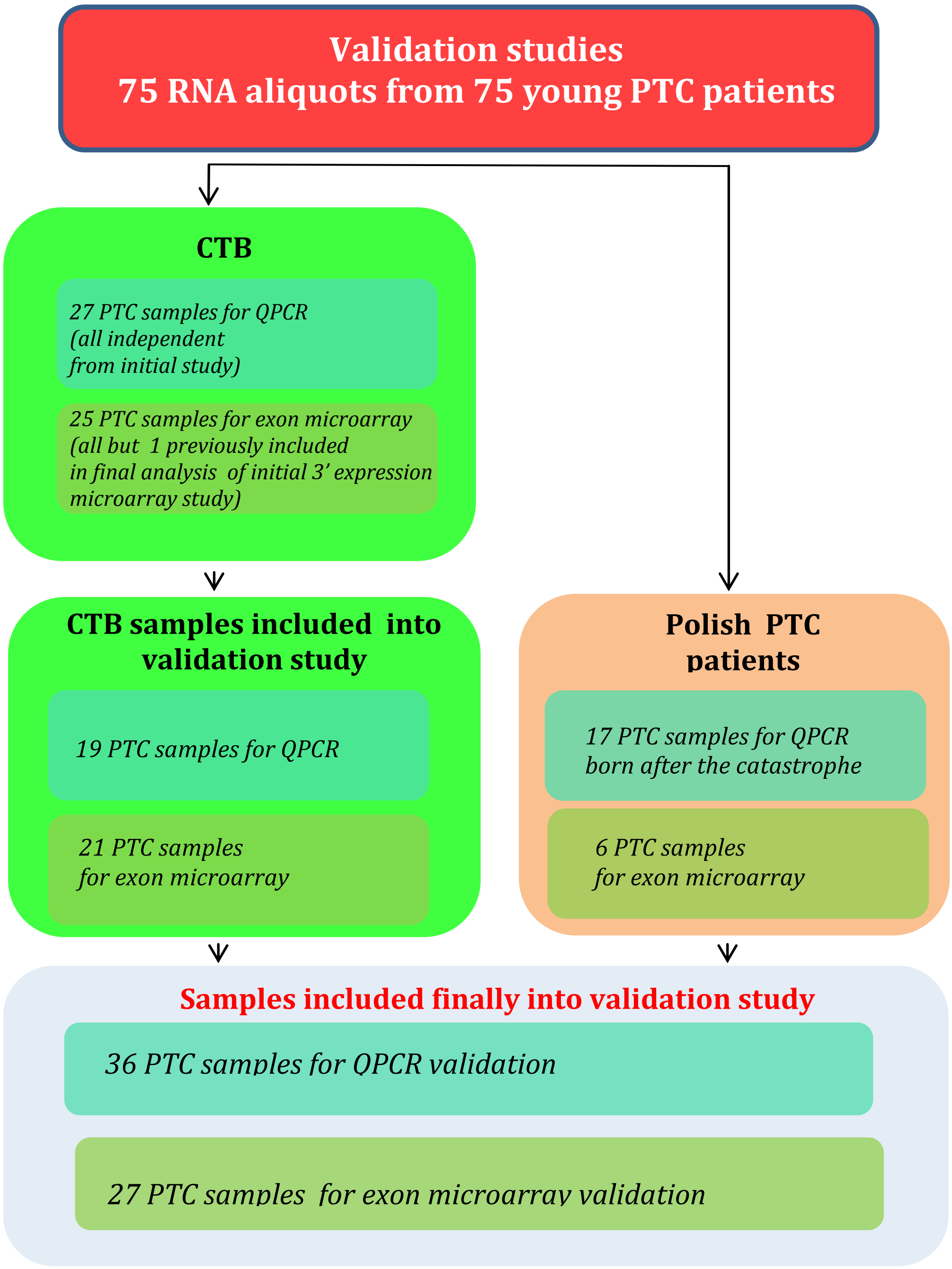
Validation studies
75 RNA aliquots from 75 young PTC patients
CTB
27 PTC samples for QPCR
(all independent
from initial study)
25 PTC samples for exon microarray
(all but 1 previously included
in final analysis of initial 3' expression
microarray study)
CTB samples included into
validation study
19 PTC samples for QPCR
21 PTC samples
for exon microarray
Polish PTC
patients
17 PTC samples for QPCR
born after the catastrophe
6 PTC samples
for exon microarray
Samples included finally into validation study
36 PTC samples for QPCR validation
27 PTC samples for exon microarray validation
