# Supplementary figures and images for: Gene signature of the post-Chernobyl papillary thyroid cancer
Source: Eur J Nucl Med Mol Imaging. 2016 Jan 26;43:1267–77. doi: 10.1007/s00259-015-3303-3 (PMC4869750; doi:10.1007/s00259-015-3303-3)

**Figure S2.**

Heat-map of 100 genes differentiating ECR and non-ECR group

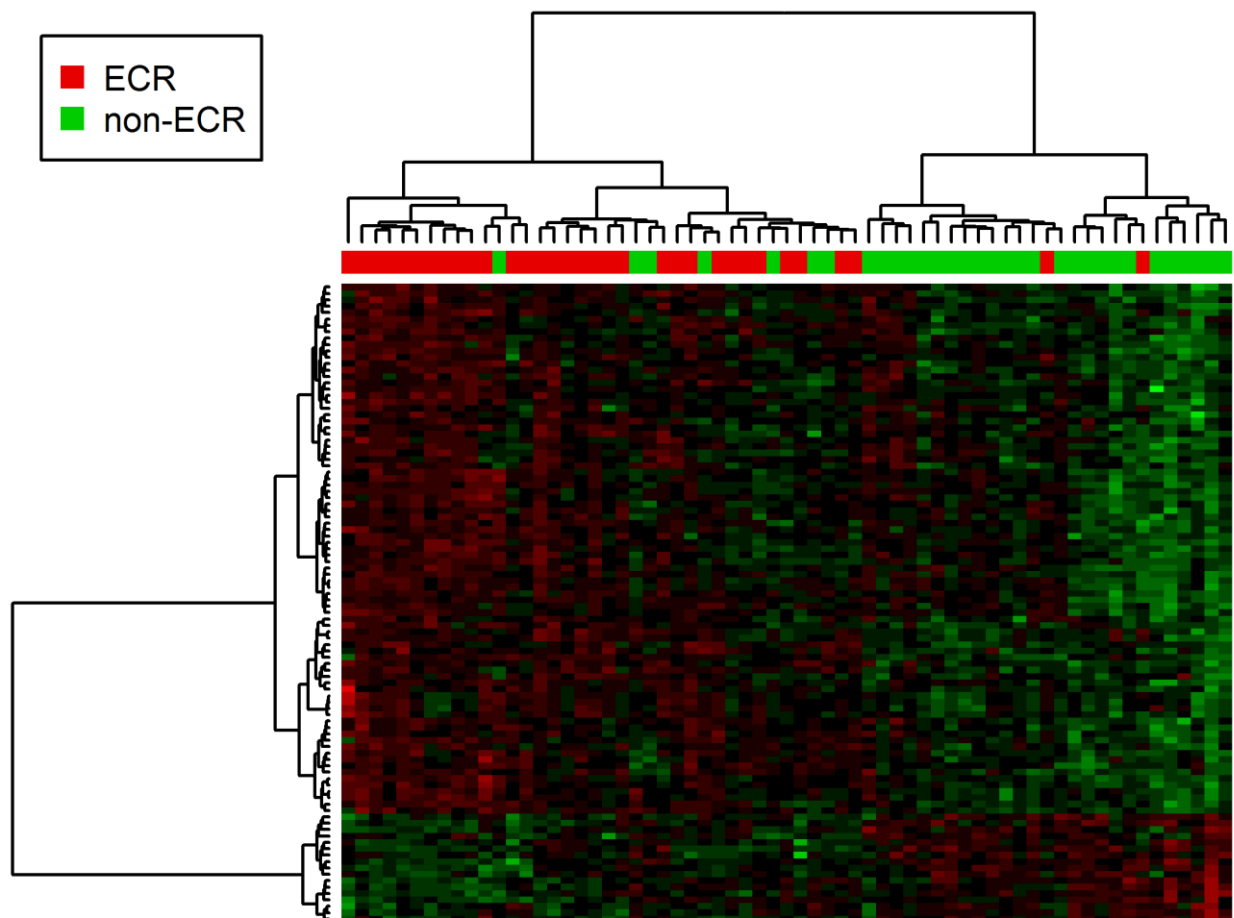

Supplement: Supplementary file 3 — (PDF 86 kb) [file 259_2015_3303_MOESM3_ESM.pdf]
