# Supplementary material for: Gene signature of the post-Chernobyl papillary thyroid cancer
Source: Eur J Nucl Med Mol Imaging. 2016 Jan 26;43:1267–77. doi: 10.1007/s00259-015-3303-3 (PMC4869750; doi:10.1007/s00259-015-3303-3)
Supplement: Supplementary file 4 — (PDF 72 kb) [file 259_2015_3303_MOESM4_ESM.pdf]

Table S1. Description of the PTC cases used for the exon microarray validation study in ECR and non-ECR PTC cases diagnosed<26 years of age.

Note that in the ECR group PTC samples were derived from patients previously investigated within the initial microarray study (all 13 cases). Non-ECR group comprised from 8 non ECR patients derived from CTB (7 included also in the microarray study) and 6 independent Polish patients <26 years of age at diagnosis, other than used in the QPCR study. For 3 Polish patients the condition to be born after 1/1/1987 was not fulfilled, thus, they should be considered “probably non-ECR”.

	**ECR**	**non-ECR**	**Statistical comparison**
Number	13	14	Not done
Female/male	8/5	12/2	ns
Age at exposition	1.9±1.0	Nd	Nod done
Age at operation	16.7±1.4	15.9±0.9	ns
**Histopathology**			
Pure classic PTC	1 (8%)	5 (36%)	
PTC follicular variant*	8 (62%)	4 (28.6%)	ns
other	4 (30%)	5 (36%)	
**Primary tumour (T stage)**			
1	7 (54%)	6 (42.9%)	ns
2	0	2 (14.3%)	
3	6 (46%)	6 (42.9%)	
**Lymph nodes (N stage)**			
0	4 (31%)	5 (35.7%)	ns
1	9 (69%)	9 (64.3%)	
**Distant Metastases (M stage)**			
0	10 (77%)	12 (86%)	ns
1	3 (23%)	2 (14%)	

*samples from CTB described as “PTC with follicular areas” were included here as PTC follicular variant

Ns - not significant
