# Supplementary material for: Gene signature of the post-Chernobyl papillary thyroid cancer
Source: Eur J Nucl Med Mol Imaging. 2016 Jan 26;43:1267–77. doi: 10.1007/s00259-015-3303-3 (PMC4869750; doi:10.1007/s00259-015-3303-3)
Supplement: Supplementary file 7 — (PDF 14 kb) [file 259_2015_3303_MOESM7_ESM.pdf]

Table S4. The three-way analyses of variance of ECR/non-ECR differences

**Effect**	**No of probesets at p< 0.001**	**No of probesets at FDR < 10%**
A. Radiation exposure-related differences in the context of the patient age at PTC diagnosis		
Exposure to Chernobyl-related radiation	347	781
Age<br><16/>16 years	20	0
Interaction<br>Exposure:Age	14	0
B. Radiation exposure-related profile in the context of the known molecular factors relevant for PTC		
Exposure to Chernobyl-related radiation	485	1531
RET rearrangment	71	13
Interaction<br>Exposure:*RET*	4	0
Exposure to Chernobyl-related radiation	209	32
BRAF mutation	443	794
Interaction<br>Exposure:*BRAF*	11	0
C. Interaction between the effect of radiation exposure and the pathological PTC variant		
Exposure to Chernobyl-related radiation	279	376
Pathology<br>Classical+follicular/Solid component	151	66
Interaction<br>Exposure:Pathology	5	0
