# Supplementary material for: Gene signature of the post-Chernobyl papillary thyroid cancer
Source: Eur J Nucl Med Mol Imaging. 2016 Jan 26;43:1267–77. doi: 10.1007/s00259-015-3303-3 (PMC4869750; doi:10.1007/s00259-015-3303-3)
Supplement: Supplementary file 8 — (PDF 344 kb) [file 259_2015_3303_MOESM8_ESM.pdf]

Table S5. Genes used in the validation of microarray data with the QPCR method (genes that confirmed the microarray data are marked with grey background). These genes are ordered according to Fold-change, defined by QPCR

Gene	Description	QPCR			Microarrays			Gene function in respect to participation in response to DNA damage and/or in regulation of ERK pathway	References
		Pvalue	FDR	Fold-Change	Pvalue	FDR	Fold-change		
RAD51AP1	RAD51 associated protein 1	1,10E-02	0,021	0,553	5,11E-05	0,036	0,58	Genetic ablation of *RAD51AP1* leads to enhanced sensitivity to chromosome aberrations upon DNA damage. *RAD51AP1* -depleted cells have deficits in recombination-based repair of a DNA double-strand break, and exhibit chromatin breaks both spontaneously and upon DNA-damaging treatment.	Dray E et al., 2011; Wiese C et al., 2007
CDK1(CDC2)	cyclin-dependent kinase 1	9,00E-03	0,021	0,673	9,40E-04	0,084	0,57	CDC2 is required for efficient 5′ to 3′ resection of double-strand break ends, and for the recruitment of both the single-stranded DNA-binding complex, RPA, and the RAD51 recombination protein. Expression of *CDC2* in fibroblasts is reduced in response to radiation. Its suppression is essential for DNA damage-induced G2 arrest.	Zhou T et al., 2006; Babtist M et al., 1996; Ira G et al., 2004
THRA	thyroid hormone receptor, alpha (erythroblastic leukemia viral (v-erb-a) oncogene homolog, avian)	2,40E-03	0,009	1,157	1,87E-04	0,059	1,32	Acts as transcriptional repressor and in this role participates in the regulation of DSB repair	Kress E et al., 2008; Yu J et al., 2006
SOCS7	suppressor of cytokine signaling 7	5,00E-04	0,004	1,215	6,35E-05	0,040	1,38	Belongs to SOX septin family. The association between septins, SOCS7 and other proteins in the DNA damage checkpoint response has been reported. SOCS7 regulates actin organization and cell cycle arrest.	Kremer BE et al., 2007;
CIC	capicua homolog (Drosophila)	1,70E-03	0,008	1,221	2,30E-05	0,034	1,26	It may encode evolutionarily conserved protein involved in hypoxia tolerance. Loss of capicua can desensitize cells to the effects of ERK pathway inhibition. CIC may represent a new member of a SOX-related HMG subfamily (OMIM database).	Dissanayake K et al., 2011; Udpa N et al., 2014
PPP1R9A	protein phosphatase 1, regulatory (inhibitor) subunit 9A	1,10E-02	0,021	1,225	1,25E-05	0,034	1,5	PP1 is a crucial component in the ATM-Chk2-p53 signaling pathway	Lu et al., 2013
PPME1	protein phosphatase methylesterase 1	1,00E-04	0,002	1,245	2,81E-04	0,063	1,19	its protein product, protein phosphatase methylesterase 1, is regarded as a key molecule that sustain, via inhibition of PP2A, the activation of ERK activity in cancer cells	Lankoff A et al., 2006; Puustinen P et al., 2009
HDAC11	histone deacetylase 11	7,00E-04	0,004	1,247	3,00E-05	0,034	1,47	It is activated by radiation. Interacts with RB-E2F to inhibit gene transcription.	Guo C et al., 2007

HDGF	hepatoma-derived growth factor (high-mobility group protein 1-like)	1,00E-02	0,021	1,275	3,92E-05	0,035	1,19	Its product is a multifunctional protein which participates in many cellular events including DNA damage repair and transcriptional regulation; its expression is related to radiosensitivity of cancer cells	Matsuyama A et al., 2007; Zhao J et al., 2011
ERBB2	v-erb-b2 erythroblastic leukemia viral oncogene homolog 2, neuro/glioblastoma derived oncogene homolog (avian)	4,30E-03	0,014	1,341	1,32E-04	0,053	1,32	A member of the EGF receptor family,as such activates MAPK pathway.	Salzano M et al., 2014
MKNK2	MAP kinase interacting serine/threonine kinase 2	0,1067	0,184	1,056	1,30E-05	0,0341	1,29	The kinase Mnk2 is a substrate of the MAPK pathway and phosphorylates the translation initiation factor eIF4E. Mnk2a downregulation by alternative splicing is a tumor suppressor mechanism.	Maimon A et al., 2014; Slentz-Kesler K et al., 2000
NRAS	neuroblastoma RAS viral (v-ras) oncogene homolog	0,1556	0,246	0,925	2,29E-04	0,0609	0,67	NRAS mutations in papillary thyroid carcinoma lead to constitutive activation of the MAPK pathway	Zou M et al., 2014
JUB	jub, ajuba homolog (Xenopus laevis)	0,2997	0,438	1,054	4,53E-05	0,0359	1,39	In mouse fibroblasts expressing Ajuba, MAPK1 (mitogen-activated protein kinase 1) activity persisted despite serum starvation and upon serum stimulation; MAPK activity was 5-fold higher than in colorectal cells	Goyal R.K et al., 1999
USP15	ubiquitin specific peptidase 15	0,5516	0,749	1,157	5,44E-05	0,0369	0,75	USP15 is a deubiquitinase, a highly conserved protein involved in the regulation of intracellular protein breakdown, cell cycle regulation and stress response	OMIM database
FAM105A	family with sequence similarity 105, member A	0,5944	0,753	1,027	1,30E-04	0,0526	0,55	Its protein product is proapoptotic protein	Mannherz O et al., 2006
MNT	MAX binding protein	0,7306	0,868	1,016	2,90E-06	0,0187	1,28	MNT is a pro-survival protein important for mitogen. Max-interacting protein MNT is up=regulated in activated lymphocytes and was found to protect lymhocytes from MYC-dependent apoptosis	Link JM & Hurlin PJ, 2014
GPX7	glutathione peroxidase 7	0,8757	0,979	0,978	1,20E-06	0,0103	0,61	GPX7 participates in response of thyrocytes to oxidative stress.	Kolypetri el al., 2014
PALM3	Paralemmin-3	0,975	1	1,049	3,38E-05	0,0341	3,42	Paralemmin 3, an ancient protein, is an isoform of paralemmin 1, a protein implicated in plasma membrane dynamics, also the invasiveness and metastatic potential of cancer cells. However, data suggest that each paralemmin has a unique function, not yet known.	Hultqvist G et al., 2012
GNA11	guanine nucleotide binding protein (G protein), alpha 11 (Gq class)	1	1	1,014	2,94E-05	0,0341	1,25	Thyrocyte specific Gq/G11- deficient mice lacked the normal proliferative thyroid response to TSH or goitrogenic diet, indicating an essential role of this pathway in the adaptive growth of the thyroid gland.	Bäck C.M et al., 2013

Rferences:

Bäck CM, Stohr S, Schäfer EA, Biebermann H, Boekhoff I, Breit A et al. TSH induces metallothionein 1 in thyrocytes via Gq/11- and PKC-dependent signaling. J Mol Endocrinol 2013; *51*:79-90.

Dissanayake K, Toth R, Blakey J, Olsson O, Campbell DG, Prescott AR et al. ERK/p90(RSK)/14-3-3 signalling has an impact on expression of PEA3 Ets transcription factors via the transcriptional repressor capicua. Biochem J 2011; *433:* 515-525.

Dray E, Dunlop MH, Kauppi L, San FJ, Wiese C, Tsai MS et al. Molecular basis for enhancement of the meiotic DMC1 recombinase by RAD51 associated protein 1 (RAD51AP1). Proc Natl Acad Sci USA 2011; *108:* 3560-3565.

Goyal RK, Lin P, Kanungo J, Payne AS, Muslin AJ, Longmore GD. Ajuba, a novel LIM protein, interacts with Grb2, augments mitogen-activated protein kinase activity in fibroblasts, and promotes meiotic maturation of Xenopus oocytes in a Grb2- and Ras-dependent manner. Mol Cell Biol 1999; *19*:4379-89.

Hultqvist G, Ocampo Daza D, Larhammar D, Kilimann MW. Evolution of the vertebrate paralemmin gene family: ancient origin of gene duplicates suggests distinct functions. PLoS One 2012;7(7):e41850. doi: 10.1371/journal.pone.0041850.

Ira G, Pellicioli A, Balijja A, Wang X, Fiorani S, Carotenuto W et al. DNA end resection, homologous recombination and DNA damage checkpoint activation require CDK1. Nature 2004; *431:* 1011-1017.

Kolypetri P and Carayanniotis G. Apoptosis of NOD.H2 h4 thyrocytes by low concentrations of iodide is associated with impaired control of oxidative stress. Thyroid 2014; *24:* 1170-1178.

Kremer BE, Adang LA and Macara IG. Septins regulate actin organization and cell-cycle arrest through nuclear accumulation of NCK mediated by SOCS7. Cell 2007; *130:* 837-850.

Kress E, Rezza A, Nadjar J, Samarut J and Plateroti M. The thyroid hormone receptor-alpha (TRalpha) gene encoding TRalpha1 controls deoxyribonucleic acid damage-induced tissue repair. Mol Endocrinol 2008; *22:* 47-55.

Link JM, Hurlin PJ. The activities of MYC, MNT and the MAX-interactome in lymphocyte proliferation and oncogenesis. Biochim Biophys Acta 2014 Apr 13. pii: S1874-9399(14)00081-9. doi: 10.1016/j.bbagrm.2014.04.004.

Lu Z, Wan G, Guo H, Zhang X and Lu X. Protein phosphatase 1 inhibits p53 signaling by dephosphorylating and stabilizing Mdmx. Cell Signal 2013; *25:* 796-804.

Maimon A, Mogilevsky M, Shilo A, Golan-Gerstl R, Obiedat A, Ben-Hur V et al. Mnk2 alternative splicing modulates the p38-MAPK pathway and impacts Ras-induced transformation. Cell Rep 2014; *24*:501-13.

Mannherz O, Mertens D, Hahn M, Lichter P. Functional screening for proapoptotic genes by reverse transfection cell array technology. Genomics 2006; *87*:665-72.

Salzano M, Vázquez-Cedeira M, Sanz-García M, Valbuena A, Blanco S, Fernández IF et al. Vaccinia-related kinase 1 (VRK1) confers resistance to DNA-damaging agents in human breast cancer by affecting DNA damage response. Oncotarget 2014; *15:*1770-8.

Slentz-Kesler K, Moore JT, Lombard M, Zhang J, Hollingsworth R, Weiner MP. Identification of the human Mnk2 gene (MKNK2) through protein interaction with estrogen receptor beta. Genomics 2000; *69*:63-71.

Udpa N, Ronen R, Zhou D, Liang J, Stobdan T, Appenzeller O et al. Whole genome sequencing of Ethiopian highlanders reveals conserved hypoxia tolerance genes. Genome Biol 2014; *15:* R36.

Wiese C, Dray E, Groesser T, San FJ, Shi I, Collins DW et al. Promotion of homologous recombination and genomic stability by RAD51AP1 via RAD51 recombinase enhancement. Mol Cell 2007; *28:* 482-490.

Yu J, Palmer C, Alenghat T, Li Y, Kao G and Lazar MA. The corepressor silencing mediator for retinoid and thyroid hormone receptor facilitates cellular recovery from DNA double-strand breaks. Cancer Res 2006; *66:* 9316-9322.

Zou M, Baitei EY, Alzahrani AS, BinHumaid FS, Alkhafaji D, Al-Rijjal RA et al. Concomitant RAS, RET/PTC, or BRAF Mutations in Advanced Stage of Papillary Thyroid Carcinoma. Thyroid 2014; *24*:1256-66.
